# Preclinical evaluation of Affibody molecule for PET imaging of human pancreatic islets derived from stem cells

**DOI:** 10.1186/s13550-023-01057-3

**Published:** 2023-12-15

**Authors:** Pierre Cheung, Julia Thorngren, Bo Zhang, Svitlana Vasylovska, Francesco Lechi, Jonas Persson, Stefan Ståhl, John Löfblom, Olle Korsgren, Jonas Eriksson, Joey Lau, Olof Eriksson

**Affiliations:** 1 Science for Life Laboratory, Department of Medicinal Chemistry, Uppsala University, Uppsala, Sweden; 2 Department of Medical Cell Biology, Uppsala University, Uppsala, Sweden; 3 Department of Protein Science, Division of Protein Engineering, KTH Royal Institute of Technology, Stockholm, Sweden; 4 Department of Immunology, Genetics and Pathology, Uppsala University, Uppsala, Sweden

**Keywords:** Affibody molecule, Stem cells, Diabetes, DGCR2, PET, Fluorine-18 chemistry

## Abstract

**Background:**

Beta-cell replacement methods such as transplantation of isolated donor islets have been proposed as a curative treatment of type 1 diabetes, but widespread application is challenging due to shortages of donor tissue and the need for continuous immunosuppressive treatments. Stem-cell-derived islets have been suggested as an alternative source of beta cells, but face transplantation protocols optimization difficulties, mainly due to a lack of available methods and markers to directly monitor grafts survival, as well as their localization and function. Molecular imaging techniques and particularly positron emission tomography has been suggested as a tool for monitoring the fate of islets after clinical transplantation. The integral membrane protein DGCR2 has been demonstrated to be a potential pancreatic islet biomarker, with specific expression on insulin-positive human embryonic stem-cell-derived pancreatic progenitor cells. The candidate Affibody molecule Z_DGCR2:AM106_ was radiolabeled with fluorine-18 using a novel click chemistry-based approach. The resulting positron emission tomography tracer [^18^F]Z_DGCR2:AM106_ was evaluated for binding to recombinant human DGCR2 and cryosections of stem-cell-derived islets, as well as in vivo using an immune-deficient mouse model transplanted with stem-cell-derived islets. Biodistribution of the [^18^F]Z_DGCR2:AM106_ was also assessed in healthy rats and pigs.

**Results:**

[^18^F]Z_DGCR2:AM106_ was successfully synthesized with high radiochemical purity and yield via a pretargeting approach. [^18^F]Z_DGCR2:AM106_ retained binding to recombinant human DCGR2 as well as to cryosectioned stem-cell-derived islets, but in vivo binding to native pancreatic tissue in both rat and pig was low. However, in vivo uptake of [^18^F]Z_DGCR2:AM106_ in stem-cell-derived islets transplanted in the immunodeficient mice was observed, albeit only within the early imaging frames after injection of the radiotracer.

**Conclusion:**

Targeting of DGCR2 is a promising approach for in vivo detection of stem-cell-derived islets grafts by molecular imaging. The synthesis of [^18^F]Z_DGCR2:AM106_ was successfully performed via a pretargeting method to label a site-specific covalently bonded fluorine-18 to the Affibody molecule. However, the rapid washout of [^18^F]Z_DGCR2:AM106_ from the stem-cell-derived islets graft indicates that dissociation kinetics can be improved. Further studies using alternative binders of similar classes with improved binding potential are warranted.

**Supplementary Information:**

The online version contains supplementary material available at 10.1186/s13550-023-01057-3.

## Introduction

Diabetes is a disease characterized by a chronic high levels of blood glucose linked to defects in insulin secretion or action pathways, often associated with a loss of a functional beta-cell mass (BCM). Currently, neither type 1 diabetes (T1D) nor type 2 diabetes (T2D) can be cured by pharmaceutical interventions. Beta-cell replacement methods have been suggested as a curative treatment for T1D. For several decades, beta-cell replacement strategies in the context of T1D have been restricted solely to transplantation of the whole pancreas or isolated human islets from brain dead donors [1]. A renewable cell source such as islets generated from human pluripotent stem cells (e.g., human embryonic stem cells (hESC) or induced pluripotent stem cells (iPSC)) has been proposed and explored to recover a functional BCM. This approach could help bypass the main challenges associated with human islet transplantation, such as frequent shortage of suitable donors and the need for extensive immunosuppressive treatments [2, 3]. Over the past decades, physiologically relevant stem-cell-derived islets (SC-islets) with a cytoarchitecture and a functionality similar to those of the adult primary islets have been successfully generated [4, 5]. Those SC-islets have demonstrated the capability to cure diabetic mice in vivo [6, 7], making them a promising alternative to human islet transplantation. Recently, in an ongoing clinical phase I/II study, three patients with T1D have been transplanted with SC-islets through an ordinary hepatic vein infusion, resulting in glucose responsive insulin secretion, reduced usage of exogenous insulin and improvement in the HbA1c levels (ClinicalTrials.gov Identifier: NCT04786262). Despite the successful generation of functional SC-islets, monitoring the transplanted SC-islets remains a challenge, especially during the critical early post-transplantation period that dictates the survivability of the grafted cells as they are prone to immune rejections [8]. Molecular imaging techniques and particularly positron emission tomography (PET) have been suggested as potential tools for monitoring the fate of islets after clinical transplantation [9]. Therefore, the identification of molecular targets restricted to newly grafted SC-islets would greatly help us monitor their fate after transplantation. The DiGeorge syndrome critical region gene 2 (DGCR2) protein has not only been identified as biomarker of mature human pancreatic beta cells [10], but also shown to be strongly expressed on insulin-positive hESC-derived pancreatic progenitor cells from immunostaining results [11].

In this study, we describe the labeling of a novel Affibody molecule Z_DGCR2:AM106_ with fluorine-18 using an innovative click chemistry approach. The resulting PET tracer [^18^F]Z_DGCR2:AM106_ was then evaluated for molecular imaging of SC-islets grafts in vitro and in vivo, as well as for biodistribution in various healthy animal species.

## Research design and methods

### Radiochemistry

The labeling process comprised three reaction steps, adapted from a previously developed method for ^18^F-radiolabeling of antibody constructs [12].

### Synthesis of [^18^F]MeTz

[^18^F]fluoride (16–20 GBq) in aqueous solution was concentrated and trapped on a Chromabond PS-HCO_3_ Shorty/45 mg cartridge (Macherey–Nagel). The [^18^F]fluoride was then reacted on the solid support with a solution of the precursor N,N,N-trimethyl-5-((2,3,5,6-tetrafluorophenoxy)carbonyl) pyridin-2-aminium trifluoromethanesulfonate 1 (10 mg) in acetonitrile (0.8 mL) which was passed over the Chromabond cartridge followed by neat acetonitrile (0.7 mL). Both solutions were passed over the cartridge at a flow rate of 0.4 mL/min. The reaction formed [^18^F]F-Py-TFP instantly at room temperature, which was then released with the flow into a Teflon tubing acting as a reservoir. After the elution was completed, the collected solution in the Teflon tubing was pushed with air over an Oasis MCX Plus Short cartridge (Waters), preconditioned with acetonitrile (3 mL), to remove unreacted cationic precursor 1. The purified solution containing [^18^F]F-Py-TFP was then delivered to a septum-equipped vial (5 mL) containing (4-(6-methyl-1,2,4,5-tetrazin-3-yl)phenyl)methanamine hydrochloride 2 (1.0 mg) in DMSO (0.1 mL) to improve its solubility.

In the second step, triethylamine (5 µL) in acetonitrile (100 µL) was added to the mixture, and the mixture was heated for 15 min at 55 °C. After diluting the mixture with aqueous trifluoroacetic acid (3 mL, 0.1%), the labeled tetrazine was isolated by semi-preparative HPLC. The column used was an ACE C18 5 µm (150 × 10 mm) column, and the eluent was a mixture of water, ethanol and TFA in a 62:38:0.01 ratio, with a flow rate of 5 mL/min. The column eluate was monitored using a UV detector (254 nm) and a radiodetector. The retention time for [^18^F]MeTz was 8.5 min. The collected HPLC fraction containing [^18^F]MeTz was reformulated in ethanol (0.5 mL) using the conventional procedure, which included dilution with water (20 mL), fixation on a Sep-Pak tC18 Plus Light Cartridge, a rinse with water (20 mL) and air prior to elution with the ethanol (0.5 mL) into a septum-equipped vial (1 ml). The ethanol was the then removed by evaporation under a stream of nitrogen gas and heat at 65 °C for 7 min, leaving the [^18^F]MeTz with only a few water droplets in the vial.

The radiochemical purity of [^18^F]MeTz was assessed by analytical HPLC using a Kinetex C18 column (2.6 µm 100 Å, 100 × 3.0 mm, Phenomenex). The eluent was a mixture of water and acetonitrile in a 75:25 ratio, with a flow rate of 0.7 mL/min. The retention time for [^18^F]MeTz was 6.6 min. The labeled tetrazine was identified by co-injection and matching retention times with an authentic reference standard (Norsk medisinsk syklotronsenter AS). The molar activity of the [^18^F]MeTz was assessed by determining the tracer concentration using the same analytical HPLC-procedure and measuring the activity in a dose calibrator.

### Synthesis of [^18^F]Z_DGCR2:AM106_

Z_DGCR2:AM106_ (containing 59 residues with the following amino acid sequence: H2N-VDNKFNKEQTHARNEILQLPNLNRMQKSAFIRSLIDDPSQSANLLAEAKKLNDAQAPKC) was manufactured by custom solid-phase peptide synthesis by New England Peptide under our request. The unique cysteine residue at position 59 at the C-terminal was added albeit not initially being part of the original Affibody molecule sequence, to enable site-specific functionalization of the peptide at this position, a trans-cyclooctene (TCO) group was conjugated to the C-terminal cysteine via maleimide chemistry, via a polyethylene glycol (PEG_3_) linker (TCO–PEG_3_–maleimide). The resulting construct TCO–Z_DGCR2:AM106_ was purified, freeze-dried and aliquoted into vials of 100 µg. The purity was > 95% as assessed by HPLC. Freeze-dried TCO–Z_DGCR2:AM106_ was first put into solution (100 µg, 14 nmol in 300 µL PBS) before adding it to a vial containing [^18^F]MeTz (2.0 ± 0.6 GBq). The mixture was then allowed to react for 7–12 min at room temperature to form [^18^F]Z_DGCR2:AM106_. The labeled Affibody molecule was then purified from unreacted [^18^F]MeTz using size exclusion chromatography with a NAP-5 column (Cytiva). After preconditioning the column with PBS and 10% ethanol (two column volumes), the product solution was loaded and then eluted with the PBS and 10% ethanol (1 mL). The ready for use [^18^F]Z_DGCR2:AM106_ was collected in the eluate (approximately 1 mL), while the [^18^F]MeTz was retained on the column.

The radiochemical purity of [^18^F]Z_DGCR2:AM106_ was assessed by analytical HPLC using a Vydac 214MS C4 column (5 µm, 300 Å, 50 × 4.6 mm, Phenomenex). The eluent was a mixture of water + 0.1% TFA and acetonitrile in a gradient running from 5 to 80% acetonitrile over 10 min, with a flow rate of 4 mL/min. The retention time for [^18^F]Z_DGCR2:AM106_ was 3.5 min. [^18^F]Z_DGCR2:AM106_ Affibody molecule was identified by co-injection and co-elution with the non-labeled TCO-conjugated Affibody molecule Z_DGCR2:AM106_.

### ELISA-based binding

Purified recombinant human DGCR2 His-tag protein (Bio-techne; #10,161-DG) was purchased for the binding studies. The freeze-dried protein was dissolved in PBS prior to coating on Pierce Copper-Coated High-Capacity Plates (Thermo Scientific) at a concentration of 10 µg/mL per well according to the manufacturer´s instruction, supplemented by a 15-min blocking step with PBS containing 1% bovine serum albumin (BSA). Wells containing PBS only were used as negative control. [^18^F]Z_DGCR2:AM106_ was incubated in each well at 5 MBq/mL for 60 min at RT with shaking. Three successive washes using PBS containing 0.05% Tween-20 were performed prior to measurement in a NaI well counter (Uppsala Imanet AB, Uppsala, Sweden).

### Human tissue and cell culture

hESC (H1 cell line, Wicell®) were differentiated into SC-islets using a previously published differentiation protocol of seven stages[4] with the following differences: H1 cells were propagated on treated human recombinant Laminin 521 (BioLamina; LN 521) in mTeSR-Plus medium (STEMCELL Technologies; #100-0274/100-275). To prepare for the differentiation step, cells were seeded at a density of 16 million cells/10 cm dish or two million cells/3.5 cm dish on plates coated with Laminin 521 in mTeSR-Plus medium with the addition of 10 µM of ROCK inhibitor Y-27632 (STEMCELL Technologies; 72,304). Differentiation was then triggered 24 h after the seeding process. During the first four stages (S1-4), cells were differentiated on adherent culture and a single cell suspension was prepared during S4:2 to generate uniform cluster formation. At stage 5:2, all SC-islets were moved to an ultra-low attachment 6-wells plate, on a rotating platform at 95 rpm. The last stages (S5-7) of the differentiation were carried out in suspension culture. During the stages S1-5, the medium was changed every day, while it was changed every second day over S6 and S7.

Pancreatic endocrine islet fractions of 47% purity were obtained from non-diabetic deceased donors within the Nordic Network for the Clinical Islet Transplantation Laboratory (Uppsala University Hospital, Uppsala Sweden) through isolation methods described by Goto et al. [13]. None of the tissues were procured from prisoners, and all the donors have provided written consent that their donated tissues may be entered into a biobank and used in medical research. The use of human tissues from Uppsala Biobank (registration #827) was approved by the Regional Ethics Board, Uppsala, Sweden (now the Swedish Ethical Review Authority) (2011/473, Ups 02–577, 2015/401), and they were anonymized, collected and treated according to local institutional and Swedish national rules and regulations. The need for informed consent was renounced by the Regional Ethics Board in Uppsala, and the study has been conducted in accordance with the Declaration of Helsinki.

The Hek293 cell line used as negative control was kindly provided by professor Di Yu (Uppsala University) and cultured in DMEM (Gibco) supplemented with 10% FBS (Gibco) and 1% PEST (Gibco). The cells were passaged using SemPro Accutase (Gibco) according to the manufacturer´s instructions.

### Frozen section autoradiography

Hek293 cells were collected using Accutase and washed with PBS before centrifugation for 5 min at 300 × g to form a pellet. Pellets of pancreatic endocrine islet fractions of 47% purity and SC-islets were obtained through sedimentation. All the obtained pellets (Hek293, human pancreatic endocrine fraction, SC-islets) were then embedded into optimal cutting temperature (O.C.T) compound (Q Path mounting media, VWR) and frozen at -80 °C. Slices of 10 µm thickness were prepared on cryotome (Cryostat NX70, ThermoFisher) before mounting on SuperFrost Plus slides (ThermoFisher) and stored at − 80 °C prior to the experiment.

Frozen sections of Hek293 cells, endocrine pancreatic islet fractions and SC-islets were incubated at RT for 60 min with PBS and 0.35 MBq/mL of [^18^F]Z_DGCR2:AM106_. A series of two 3-min cold washes in PBS with 1% BSA and one 1-min wash in deionized water were performed at the end of the incubation time. Samples were air-dried and left for exposure on the BAS-IP storage phosphor screen (Cytiva) for 120 min. 20 µL droplets of reference radioactive solution on absorbent paper, cross-measured in a NaI well counter (Uppsala Imanet AB, Uppsala, Sweden) were also included to enable quantification. The resulting digital image readout was obtained using an Amersham Typhoon storage phosphor imager (GE healthcare). The resulting autoradiography images were analyzed using ImageJ with manual delineation (details of the analysis are given in supplementary methods).

### PET/MRI/CT of mice with intramuscular SC-islet grafts

A total of n = 4 NMRI nu/nu mice (Taconic M&B) with weight of 20–30 g were transplanted with 200–800 SC-islets in the abdominal and/or neck muscle. Mice were anesthetized with isoflurane before exposition of the muscle. SC-islets were packed in a butterfly needle and implanted into the muscle layer. Subcutaneous carprofen 5 mg kg-1 (Rimadyl Vet) was used as analgesic. Approximately four weeks after transplantation, for each mouse, a magnetic resonance imaging (MRI) (517 ms TR, 12.6 ms TE, 17 slices, 1 Th, 0.1 Gap, 80 × 60 mm FOV, 0.313 mm resolution, 4 NEX, 6 min 30 s acquisition time) or computer tomography (CT) (50 kVp, 610 µA, 480 proj, 1:4 binning, 251 × 251 × 251 µm voxel size) acquisition for attenuation and anatomical localization was first performed. 400 kBq/g of [^18^F]Z_DGCR2:AM106_ was subsequently administered through the lateral tail vein. A dynamic PET scan of 60 min was then acquired and reconstructed with the Tera-Tomo 3D method (6 × 10’’; 4 × 1’; 1 × 5’; 5 × 10’ frame, 0.4 × 0.4x0.4 mm voxels, 212 × 212y 235z matrix size, 4 iterations). All the sequences described previously were performed on a nanoScan PET/MRI (for the MRI and PET sequences) or nanoScan SPECT/CT scanner (for the CT sequences) both from Mediso. Euthanasia of the mice was conducted 60 min post-injection by cervical dislocation. The intramuscular grafts were then collected alongside a piece of reference muscle for measurement in a NaI well counter (Uppsala Imanet AB, Uppsala, Sweden).

The time-activity curve (TAC) derived from each frames of the PET scan were used as inputs to calculate the volume of distribution (Vt) via the Logan plot model using the kinetic modeling tool PKIN (PMOD technologies LLC). The left ventricle of the heart was used as the blood input function and corrected for the plasma to blood ratio as well as the metabolites parent fraction.

### Immunofluorescent staining

SC-islets were fixed in 4% paraformaldehyde (PFA) at RT for 20 min and frozen. Muscle grafts were fixed overnight in 4% PFA at 4˚C, followed by incubation in 15% sucrose in PBS during 2–3 h and then 30% sucrose in PBS overnight at 4˚C and frozen. Cryosections of SC-islets or grafts were prepared with a thickness of 10 µm. Sections were blocked with 3% donkey serum (Jackson Immunoresearch Laboratories) in PBS for 30 min followed by staining with the primary polyclonal rabbit antibodies against DGCR2 (1:400, Thermo Fisher Scientific, #PA5-51,474), and polyclonal guinea pig antibodies against human insulin (1:400, Fitzgerald, Acton, #20-IP30) at 4 °C overnight. The next day, the following secondary antibodies diluted 1:300: Alexa Fluor 488-conjugated donkey anti-rabbit (Jackson Immunoresearch Laboratories) and Alexa Fluor 594-conjugated donkey anti-guinea pig (Jackson Immunoresearch Laboratories) were incubated for 1 h at RT. The nuclei were stained with DAPI. All images were obtained using a laser scanning confocal microscope Zeiss LSM 780.

### Biodistribution of [^18^F]Z_DGCR2:AM106_ in rats and pigs

A MRI acquisition for attenuation and localization was first performed on n = 2 rats (Sprague Dawley, males, weight = 250–300 g) using the following parameters: 438.2 ms TR, 3.9 ms TE, 235 slices, 5 Th, 0 Gap, 80 mm FOV, 0.5 mm resolution, 2 NEX, 20 min acquisition time. Subsequently, 50 kBq/g of [^18^F]Z_DGCR2:AM106_ (equivalent to a peptide dose of 10 µg) dissolved in PBS and maximum 10% ethanol was injected intravenously through the tail vein of each rat. A dynamic PET imaging of 90 min was then acquired using the following parameters: 3 bed positions of 2 × 1.4’, 2 × 3.2’, 1 or 2 × 10’, 0.4 × 0.4 × 0.4 mm voxels, 212 × 21 2 × 583 matrix size. All of the sequences described previously were performed using a nanoScan PET/MRI scanner (Mediso).

A total of n = 2 pigs (Yorkshire x Swedish Landrace x Hampshire, males, weight = 25–30 kg) were anesthetized and intubated according to a standardized procedure previously described [14]. A CT acquisition for attenuation and localization was first performed (100 kV, 80–400 mA, noise index 10, rotation 0.50, full spiral, slice thickness 3.75 mm, pitch 0.98:1, recon diameter 50 mm) before injecting approximately 30 MBq/kg of [^18^F]Z_DGCR2:AM106_ (equivalent to a peptide dose of 50 µg) dissolved in PBS. A dynamic PET imaging of 90 min was then acquired using the following parameters: 33 frames of 12 × 10, 6 × 30, 5 × 120, 5 × 300 and 5 × 600 s, VPFX-S, 3 i/16 s, 256 × 256 × 89 pixels, 3 mm post-filter, 500-mm-diameter zoom. Once the PET/CT dynamic scan was finished, a pancreas contrast CT acquisition using 3 mL/kg Omnipaque (GE Healthcare), bolus tracking with 100 HU threshold and region of interest placed in aorta, 15-s monitor delay for arterial phase, and 60-s delay for venous phase with 3.5 mL/s flow was performed. All of the sequences described previously were performed using a Discovery MI PET/CT scanner (GE Healthcare).

The resulting PET data were analyzed via manual segmentation using the PBAS modeling tool (PMOD technologies LLC). The uptake in kBq/cc was converted to standardized uptake values (SUV) by correcting for the administered radioactivity and the weight of each animal.

## Results

### Fluorine-18 radiolabeling of Z_DGCR2:AM106_

**Fig. 1:**
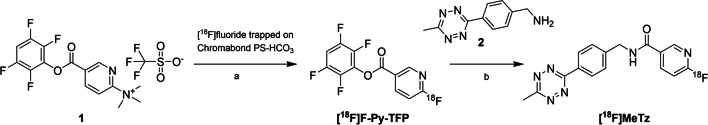
Synthesis of [^18^F]MeTz

**Fig. 2:**
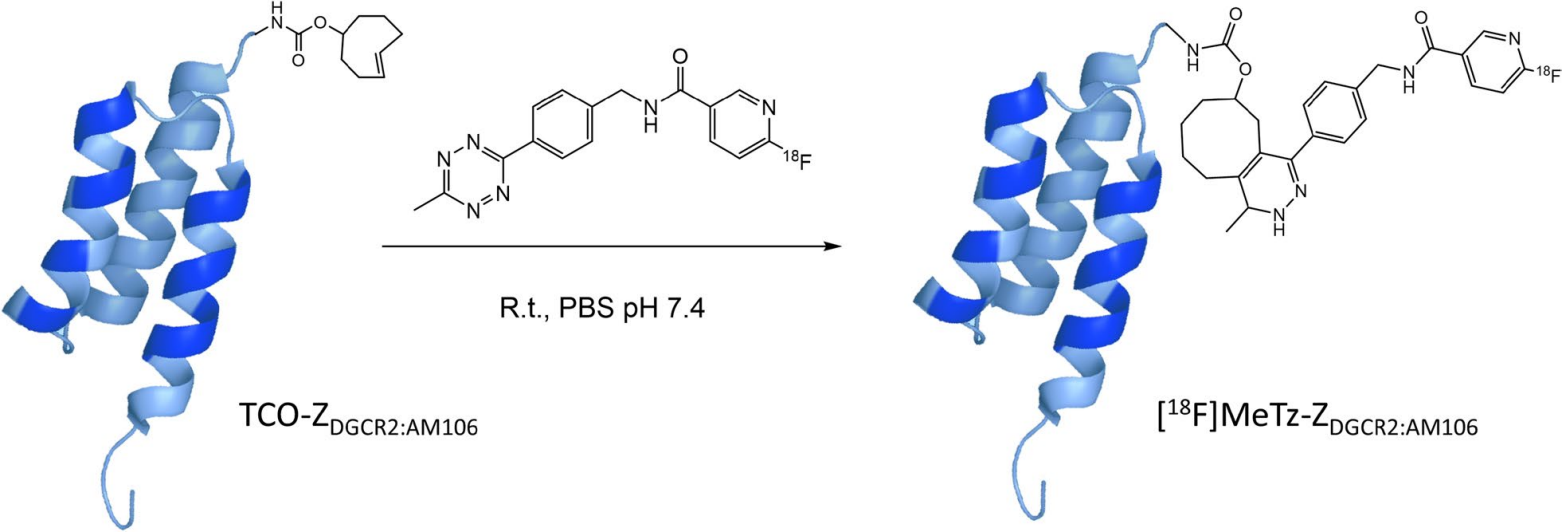
Synthesis of [^18^F]Z_DGCR2:AM106_

In the first step, an activated ester, [^18^F]F-Py-TFP, was synthesized directly on a QMA cartridge containing trapped [^18^F]fluoride by elution with a solution of the nicotinic trimethylammonium precursor **1**, bypassing the conventional fluorine-18 drying process. The precursor was removed using a cation exchange cartridge, after which the ester was coupled with a tetrazine amine forming [^18^F]MeTz. The tetrazine [^18^F]MeTz was obtained with a radiochemical yield of 18 ± 6%, a radioactivity yield of 2.0 ± 0.5 GBq and a radiochemical purity of > 95% (Fig. 1). The molar activity at the end of synthesis was 180 ± 50 GBq/µmol. The radiolabeling of [^18^F]Z_DGCR2:AM106_, was straightforward and involved adding the Affibody molecule TCO–Z_DGCR2:AM106_ in small volume of PBS to the [^18^F]MeTz suspended in a few droplets of water. The reaction proceeded at room temperature for 7–12 min (Fig. 2).

**Fig. 3:**
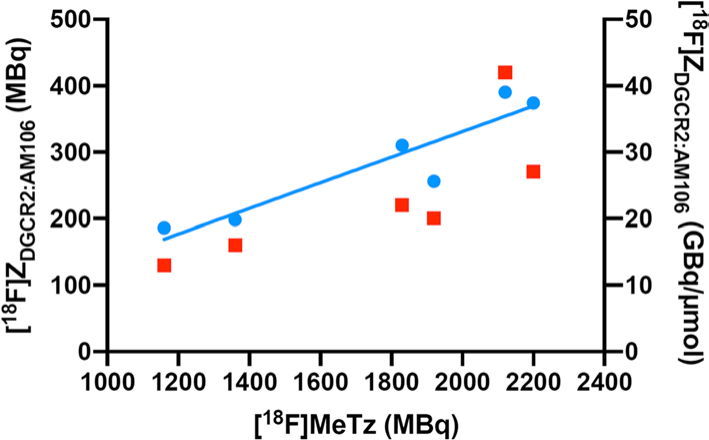
In all reactions, a consistent amount of TCO–Z_DGCR2:AM106_ was used. As the amount of [^18^F]MeTz was increased, both the molar activity (red squares) and radioactivity yield (blue dots) of the resulting [^18^F]Z_DGCR2:AM106_ also increased. This suggests that a portion of the TCO groups remained unoccupied and available for further reactions, possibly contributing to the observed increase in yield

The ready-to-use Affibody molecule [^18^F]Z_DGCR2:AM106_ formulated in approximately 1 mL of PBS and 10% ethanol was obtained with a radiochemical yield of 16 ± 2% (*n* = 7) based on [^18^F]MeTz, a radioactivity yield of 295 ± 100 MBq and a radiochemical purity of > 99% at the end of synthesis. The product showed 97.9% radiochemical purity one hour after the end of synthesis, with an initial radioactivity concentration of 250 MBq/mL, demonstrating good stability in the formulation. The molar activity of [^18^F]Z_DGCR2:AM106_ at the end of synthesis was 23 ± 9 GBq/µmol. The molar activity was determined by dividing the activity in the formulated product by the amount of TCO–Affibody used in the labeling reaction; hence, it is an estimate and can be described as the apparent molar activity. Throughout all syntheses, the amount of TCO–Z_DGCR2:AM106_ used remained constant. However, as more [^18^F]MeTz was added to the reactions, both the molar activity and radioactivity yield of the [^18^F]Z_DGCR2:AM106_ product increased (Fig. 3). This suggests that some TCO groups remained intact and contributed to the observed increase in yield.

### Copper-coated plates ELISA-based binding of [^18^F]Z_DGCR2:AM106_

**Fig. 4:**
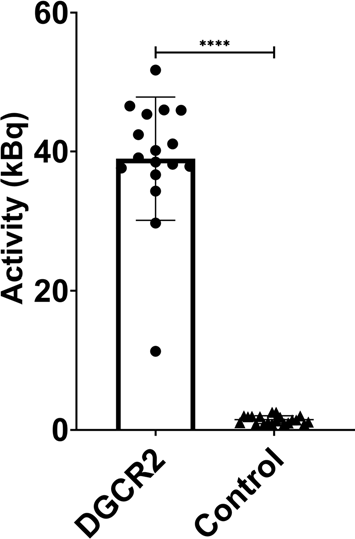
Binding signal of [^18^F]Z_DGCR2:AM106_ to recombinant human DGCR2 protein and negative control. The measured binding signal of [^18^F]Z_DGCR2:AM106_ to the recombinant human DGCR2 in kBq was significantly higher compared with the negative control (*p* < 0.0001)

[^18^F]Z_DGCR2:AM106_ showed a significantly higher binding to the purified recombinant human DGCR2 protein (38.96 ± 8.88 kBq, *n* = 17) compared with the negative control (1.46 ± 0.57 kBq, n = 20) (p* < 0.0001) using the unpaired *t*-test (Graphpad Prism 8.4.3) (Fig. 4).

### Frozen section autoradiography

**Fig. 5:**
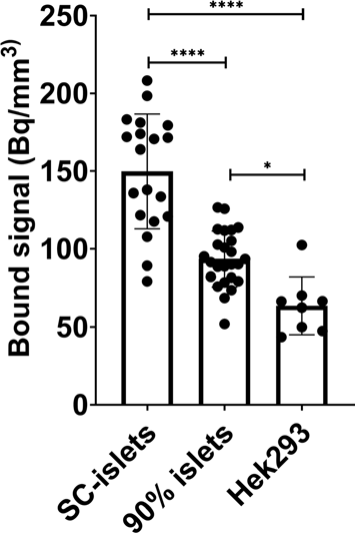
Binding signal of [^18^F]Z_DGCR2:AM106_ to frozen sections of SC-islets, isolated human pancreatic islets of 90% purity and Hek293 cells

Frozen sections of SC-islets (uptake signal = 149.8 ± 36.98 Bq/mm^3^, *n* = 19) showed a significantly higher binding of [^18^F]Z_DGCR2:AM106_ compared with frozen sections of 90% purity human pancreatic islets (uptake signal = 93.59 ± 17.91 Bq/mm^3^, *n* = 25) (p < 0.0001) and frozen sections of Hek293 negative control cells (uptake signal = 63.64 ± 18.63 Bq/mm^3^, *n* = 8) (*p* < 0.0001). The uptake signal of the 90% purity human pancreatic islets were also significantly higher than the uptake signal of the Hek293 cells (*p* < 0.05) (Fig. 5). The statistical analysis was performed using one-way ANOVA with Tukey’s multiple comparison test (Graphpad Prism 8.4.3).

### PET/MRI/CT of mice with intramuscular SC-islet grafts

**Fig. 6:**
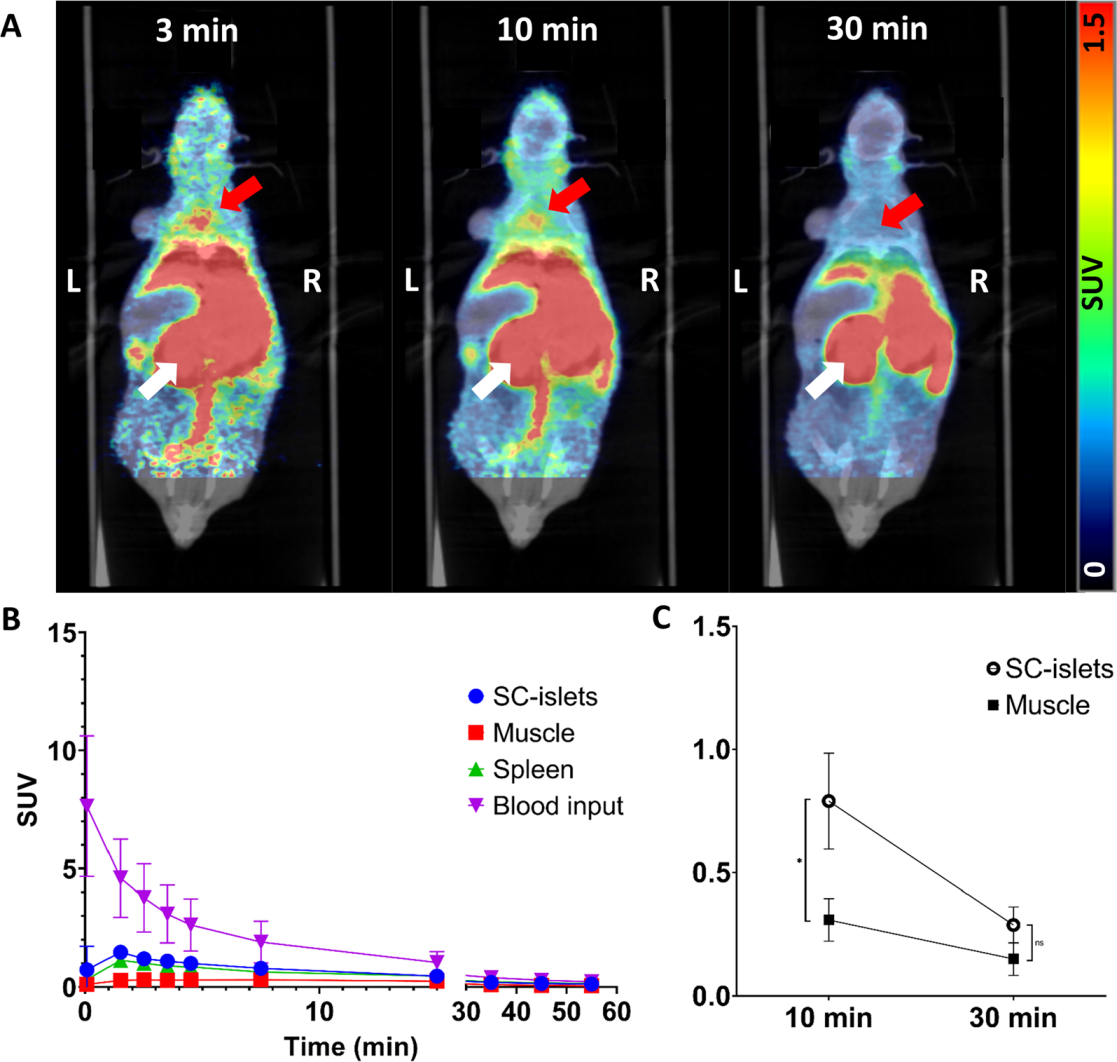
**A** Representative coronal PET/CT image of a mouse at 3, 10 and 30 min after venous administration of [^18^F]Z_DGCR2:AM106_. White arrow indicate the kidneys and red arrows indicate the engraftment site of the SC-islets. **B** TAC of the [^18^F]Z_DGCR2:AM106_ Affibody molecule. **C.** SUV of the SC-islets and reference muscle at 10 and 30 min p.i of the [^18^F]Z_DGCR2:AM106_ tracer (p* < 0.03)

The SC-islets grafted in the neck were clearly identifiable in the early PET frames after administration of [^18^F]Z_DGCR2:AM106_, but the signal is quickly fading out at later time-points (Fig. 6A-B), with a significant difference of SUV between the intramuscular SC-islets grafts (SUV = 0.79 ± 0.19) and muscle tissue (SUV = 0.31 ± 0.09) at 10 min p.i (*p* < 0.03) (Fig. 6C). The SC-islets grafted in the abdominal area could not be defined due to the large spill-over signal from the kidneys.

**Fig. 7:**
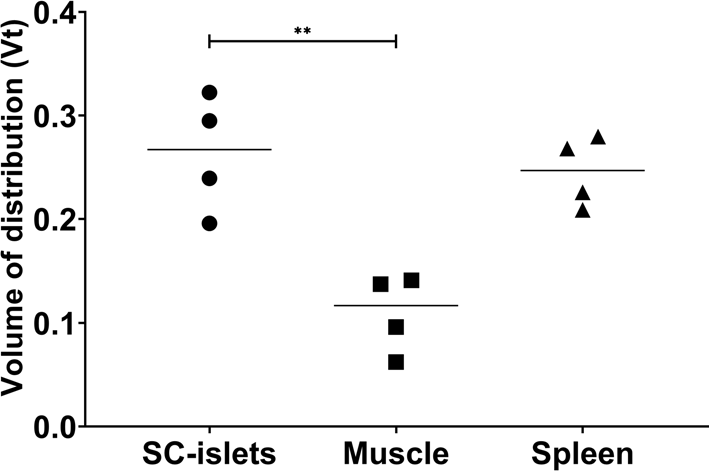
Vt for the SC-islets grafts, muscle and spleen calculated from the Logan plot model

The calculated Vt from the Logan plot, defined as the ratio between the concentration of radiotracer in the tissue of interest to that of it in plasma, was significantly higher in the SC-islets grafts (Vt = 0.26 ± 0.06) than the muscle (Vt = 0.11 ± 0.04) (Fig. 7).

### Immunofluorescence staining of the SC-islets

**Fig. 8:**
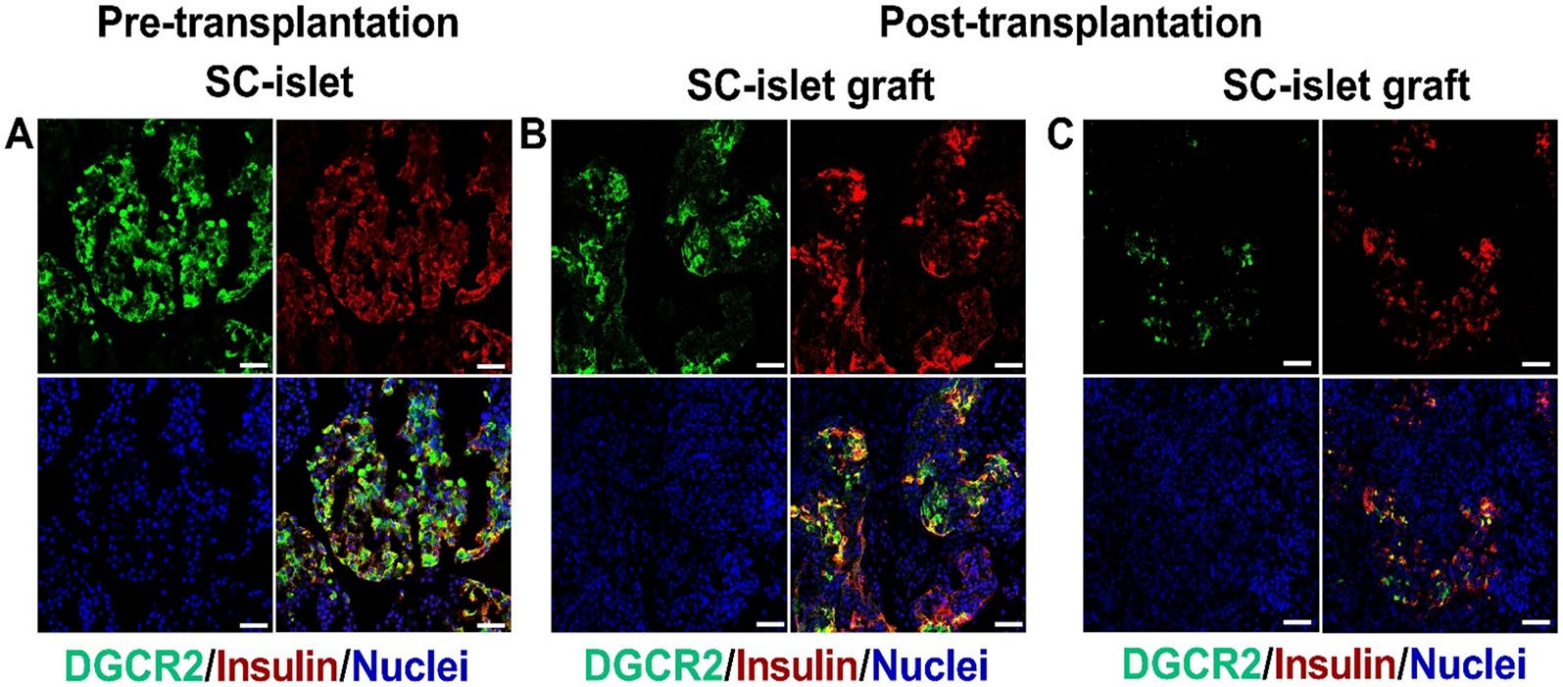
Expression of DGCR2 and insulin of SC-islets **A.** Before transplantation, SC-islets immunostained for DGCR2 (green), insulin (red) and nuclei (blue) showed co-localization of DGCR2 and insulin. After transplantation, the expression of DGCR2 (green) was observed in retrieved grafts of SC-islets. The expression of DGCR2 was observed to co-localize with the expression of insulin in SC-islet grafts retrieved from both **B.** abdominal muscle site and **C.** neck muscle site. Scale bar 50 µm

The DGCR2 immunofluorescence staining showed that SC-islets expressed DGCR2 protein before transplantation, which was observed to co-localize with insulin-positive cells (Fig. 8A). Four weeks post-transplantation, the expression of DGCR2 remained present and in co-localization with the expression of insulin in the SC-islet grafts (Fig. 8B and C), confirming the presence of DGCR2 on insulin secreting cells before and after transplantation.

### Biodistribution in rats and pigs

**Fig. 9:**
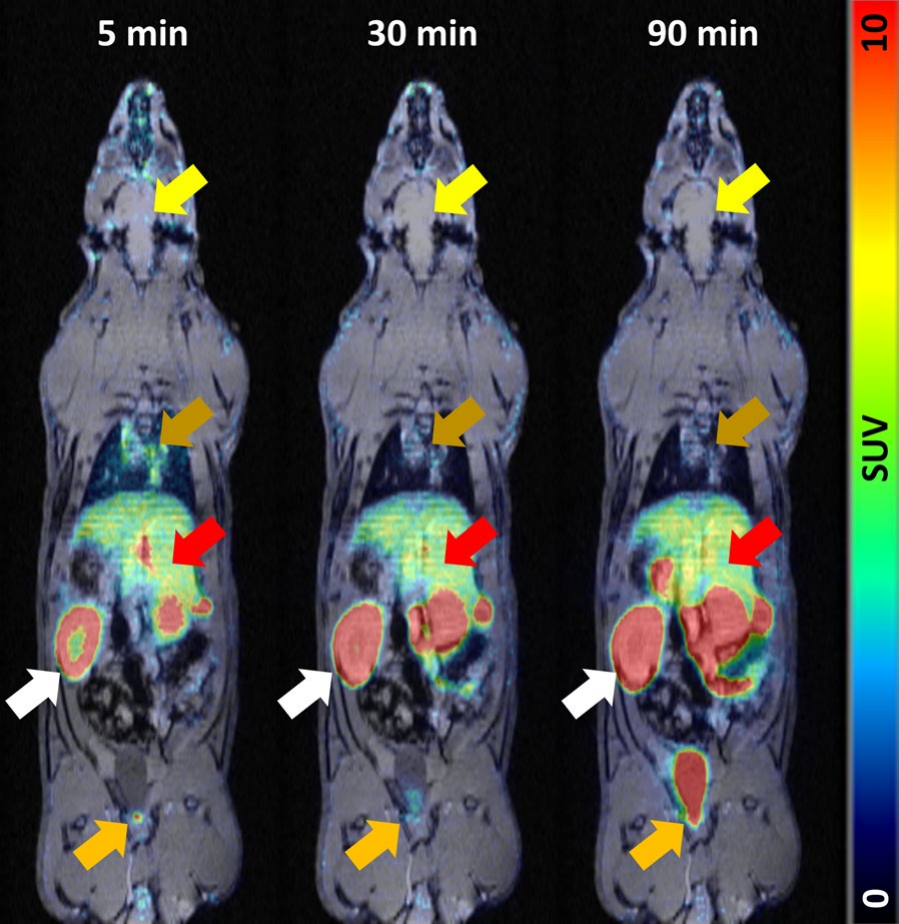
PET/MRI coronal images of a representative rat at 5, 30 and 90 min p.i of [^18^F]Z_DGCR2:AM106_ through the tail vein. White arrows indicate the kidneys, orange arrows indicate the urinary bladder, red arrows indicate the liver, brown arrows indicate the heart and yellow arrows indicate the brain

The dynamic biodistribution of [^18^F]Z_DGCR2:AM106_ in rats over the 90-min PET/MRI demonstrated mainly a renal clearance peaking at 60 min p.i, along with low liver uptake and low tissue background (Fig. 9A), similar to the results obtained from the endpoint biodistribution through the measurement of collected organs (Fig. 9B). No accumulation of [^18^F]Z_DGCR2:AM106_ could be observed in the native pancreas.

**Fig. 10:**
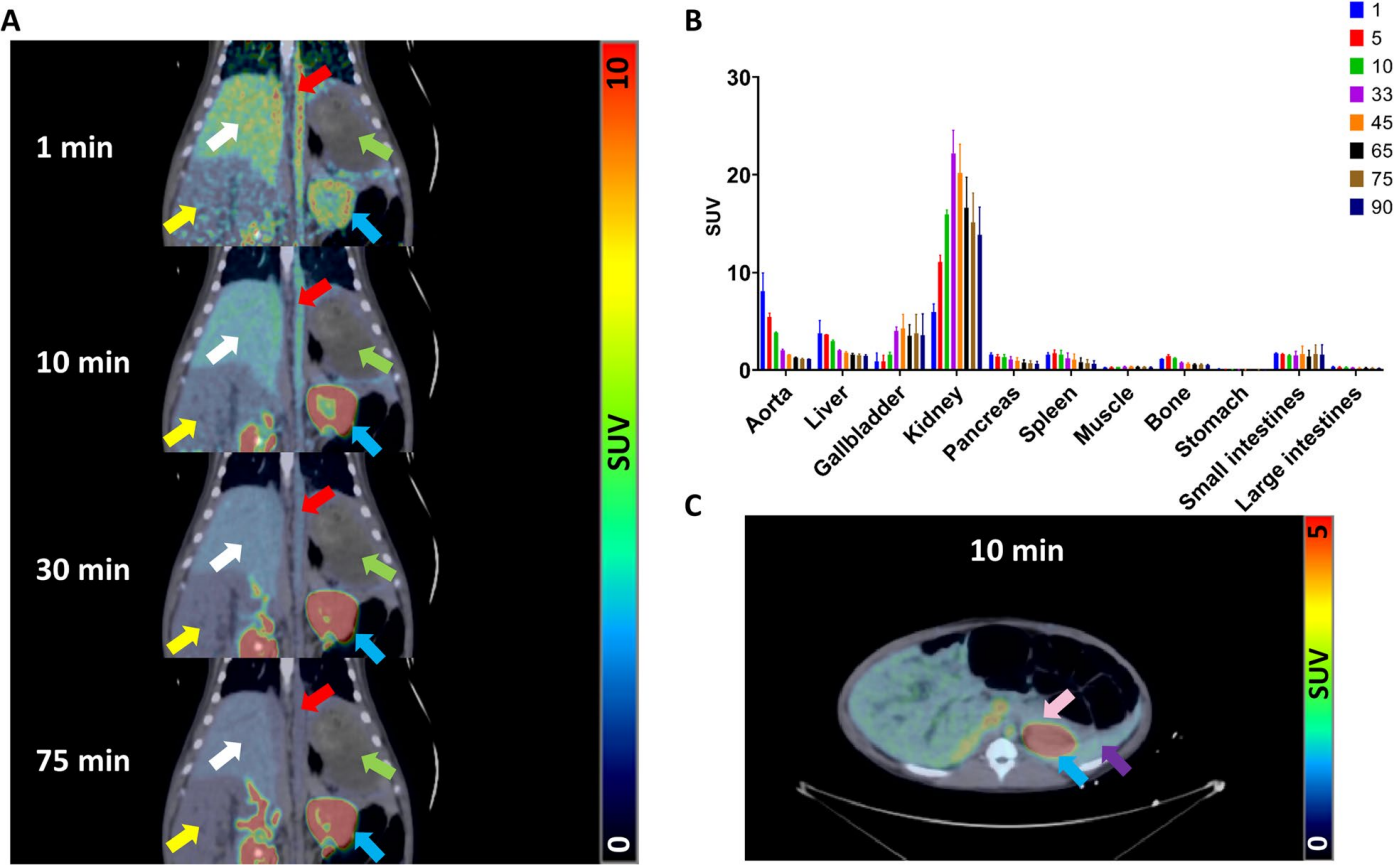
**A** PET/CT coronal images of a representative pig at 1, 10, 30 and 75 min after venous administration of [^18^F]Z_DGCR2:AM106_. White arrows indicate the liver, yellow arrows indicate the small intestines, red arrows indicate the vena cava, green arrows indicate the stomach and blue arrows indicate the kidney. **B** TAC bar graph of [^18^F]Z_DGCR2:AM106_ in various tissues. **C.** PET/CT transversal image of the pig 10 min after venous administration of [^18^F]Z_DGCR2:AM106_. Blue arrow indicates the kidney, purple arrow indicates the spleen and pink arrow indicates the pancreas

Early uptake of the radioactive tracer could be observed in the pig liver, but was gradually cleared over the duration of the scan (Fig. 10A). A strong renal clearance could be observed with a peak at 30 min p.i. which persisted throughout the examination along with a marginal uptake in the other abdominal organs (Fig. 10B). Defining the pancreas and the spleen within the pig was complicated due to the high proximity and high signal derived from the kidneys causing a signal spill-over (Fig. 10C). Clearance from the blood and non-excretory organs seemed to have occurred to a sufficient degree starting from 60 min post-administration of [^18^F]Z_DGCR2:AM106_.

## Discussion

DGCR2 is a putative excellent target for molecular imaging of mature beta cells as highlighted by previous studies [10, 11]. In the present study, we demonstrate that DGCR2 is expressed in SC-islets similar to adult primary islets, with significant co-localization with insulin, and with stable expression over several weeks following engraftment in either the abdominal or neck muscle. The Affibody molecule Z_DGCR2:AM106_ was labeled with fluorine-18, recognized for its low energy range thus offering better resolution, especially crucial for smaller structures such as pancreatic islets. The labeling was performed using a novel TCO–tetrazine click chemistry approach. Importantly, the TCO–tetrazine reaction involving the TCO-conjugated peptide can be performed using mild conditions (i.e., neutral pH and room temperature), therefore minimally affecting the peptide tertiary structure or binding capability. Introducing covalently bonded fluorine-18 via Inverse Electron Demand Diels–Alder (IEDDA) reactions offers multiple potential advantages when labeling large molecules such as proteins and peptides. This method facilitates site-specific labeling of the molecule, as the label is attached exclusively to a specific group on the molecule eliminating non-specific labeling. Such precision can help maintain the biological activity and function of the labeled molecule. Covalent bonding can also improve the stability of the labeled molecule. Non-covalently bound labels can be more prone to dissociation, which can affect the specificity of the labeled compound. In contrast, covalently bound labels are more stable and are less likely to dissociate or degrade, which can make the labeled molecule more reliable for use in imaging. With this in mind the Affibody molecule DGCR2, functionalized with a TCO group, was covalently radiolabeled with fluorine-18 using [^18^F]MeTz via a IEDDA reaction. Similar to radiolabeling of large molecules with radiometals using chelating chemistry the separation of the TCO– Z_DGCR2:AM106_ starting material from the [^18^F]Z_DGCR2:AM106_ was not feasible. Considering that the Affibody molecule was site-specifically functionalized with only one TCO group per molecule, the ratio between the radiolabeled tetrazine compound and the number of available TCO groups was concluded to be near equimolar, with a ratio between 1:1 and 1.8:1 based on the estimated concentrations of the two reactants. The ability to perform the fluorine-18 labeling and form covalent bonds at near equimolar conditions demonstrates the extreme efficiency of the IDDEA reaction. In this study, the amount of precursor material used was reduced by a factor of 1,000 compared to the typical conditions required for nucleophilic substitution reactions with [^18^F]fluoride, which usually require micromolar quantities, enabling the labeling of [^18^F]Z_DGCR2:AM106_ with high apparent molar activity without the need for separation from its precursor. Additionally, [^18^F]Z_DGCR2:AM106_ demonstrated robust stability in human plasma (parent fraction > 90%) up to 90 min and therefore excluding any risks of stability issues (Additional File 2: Figure S1).

The binding capability of [^18^F]Z_DGCR2:AM106_ was first evaluated in vitro toward the recombinant human DGCR2 using an ELISA-based method and further cross-checked via bio-layer interferometry (Additional File 3: Figure S2). The binding was found to be maintained, suggesting the labeling process did not affect the binding capability of [^18^F]Z_DGCR2:AM106_ to DGCR2. Additionally, the frozen section autoradiography showcased a significantly higher binding of [^18^F]Z_DGCR2:AM106_ to frozen sections of SC-islets compared with frozen sections of human pancreatic endocrine islet fractions of 90% purity and Hek293 cells, suggesting that DGCR2 could be a more suitable biomarker for stem-cell-derived human pancreatic islets compared to native pancreatic islets.

Interestingly, in vivo visualization using [^18^F]Z_DGCR2:AM106_ was demonstrated in the pilot study using mice grafted with SC-islets, with confirmed DGCR2 expression. However, the uptake signal of [^18^F]Z_DGCR2:AM106_ within the SC-islets grafts was only apparent during the early frames of the PET scan (≈ 10 min p.i.). Thus, this observation of signal in the grafts may be explained either by rapid, increased tracer delivery early during the PET examination, perhaps due to a perfusion effect. Secondly, it may also be that [^18^F]Z_DGCR2:AM106_ binds reversibly to DGCR2, with a modest dissociation rate causing a decrease in uptake signal early during the scan.

To assess the biodistribution of [^18^F]Z_DGCR2:AM106_ and define an imaging window with optimal contrast, PET imaging was performed in healthy rats, as well as larger animals such as pigs who usually exhibit strong translational value and high homology to humans. The biodistribution in rats highlighted the strong renal clearance of [^18^F]Z_DGCR2:AM106_, expected to a certain extent from a peptide compound with low lipophilic properties. Similarly, the biodistribution of [^18^F]Z_DGCR2:AM106_ in pigs showed excretion of the tracer mainly through the kidneys. Low level of hepatic uptake could be observed in both rats and pigs likely due to the presence of some tetrazine metabolites, but are quickly cleared out. The TAC highlighted a peak in the renal clearance at 30 min p.i., emphasizing the rapid washout property of the Affibody molecule which is a desirable feature for an imaging probe to obtain optimal contrast during scanning. The close proximity of the kidneys with the pancreas and spleen poses a high risk of spill-over signal and thus highly impairs the possibility of native beta-cell imaging, but should not be a limitation for imaging ectopic transplanted grafts. Lastly, no uptake of [^18^F]Z_DGCR2:AM106_ within the brain could be detected, suggesting that the tracer is unable to cross the blood–brain barrier.

The exact function of DGCR2 is unknown, but the deletion of the associated gene causes 22q11.2DS (previously known as DiGeorge syndrome), a developmental disorder. Interestingly, 22q11.2DS was recently shown to also be associated with both defects in the immune system, obesity and Type 2 Diabetes [15, 16]. Furthermore, DGCR2 is localized in a region on chromosome 22, which is the home of several long non-coding RNA sequences that have been identified in pancreatic beta cells, as well as islet enhancer clusters [17, 18]. DGCR2 expression has also been independently reported on extracellular vesicles released from beta cells [19].

A final reflection is that the selection of lead candidate Z_DGCR2:AM106_ could be improved, notably by modifying the selection criteria from the affinity maturation process of the Affibody molecules [20] by selecting affinity binders primarily focusing on an optimal dissociation rate. We could also lower the temperature selection threshold for the identified Affibody molecules, as the TCO–tetrazine click chemistry radiolabeling method described in this paper can be performed in mild and non-denaturing conditions.

In summary, we have presented the radiolabeling method based on TCO–tetrazine click chemistry to produce the highly stable [^18^F]Z_DGCR2:AM106_ for PET imaging of human DGCR2. [^18^F]Z_DGCR2:AM106_ exhibited retained binding capacity to both recombinant human DGCR2 and SC-islets sections in addition to a suitable biodistribution profile in multiple animal species. Successfully transplanted SC-islets grafts in immunodeficient mice were confirmed to retain expression of DGCR2 for many weeks, showing significant overlap with insulin staining. Lastly, the SC-islets could be visualized in vivo using [^18^F]Z_DGCR2:AM106,_ although the Affibody molecule demonstrated rapid washout from the target tissue.

## Conclusion

This study provided robust evidence that DGCR2 is a promising marker for molecular imaging of stem-cell-derived human pancreatic islets. The DGCR2 targeting fluorine-18 labeled Affibody molecule [^18^F]Z_DGCR2:AM106_ successfully visualized SC-islets in vivo, but the sensitivity could potentially be optimized by improving the retention of the tracer in the target tissue. Future studies using alternative Affibody molecule high-affinity binders from the same class as Z_DGCR2:AM106_ may be explored for progress in molecular imaging of DGCR2.

### Supplementary Information

**Additional file 1. Supplementary methods:** Materials and methods used for plasma meabolites analysis, Bio-Layer Interferometry and quantification for frozen section autoradiography.

**Additional file 2. Fig S1:** In vitro stability of [^18^F]Z_DGCR2:AM106_ in human plasma at t = 0 min and t = 90 min.

**Additional file 3. Fig S2:** Bio-Layer interferometry result of TCO-Z_DGCR2:AM106_ at 100 nM. Blue arrow indicates binding of DGCR2 to the interferometer, red arrow indicates binding of TCO-Z_DGCR2:AM106_ interferometer.

## Data Availability

The datasets generated during and/or analyzed during the current study are available from the corresponding author on reasonable request.

## Abbreviations

BCM: Beta-cell mass
BSA: Bovine serum albumin
CT: Computed tomography
DGCR2: DiGeorge syndrome critical region gene 2
hESC: Human embryonic stem cells
IEDDA: Inverse electron demand Diels–Alder
iPSC: Induced pluripotent stem cells
MRI: Magnetic resonance imaging
O.C.T: Optimal cutting temperature
PET: Positron emission tomography
PFA: Paraformaldehyde
PPP: Preclinical PET-MRI platform
SC-islets: Stem-cell-derived islets
SUV: Standardized uptake value
T1D: Type 1 diabetes
T2D: Type 2 diabetes
TAC: Time-activity curve
Vt: Volume of distribution
