# Supplementary material for: Preclinical evaluation of Affibody molecule for PET imaging of human pancreatic islets derived from stem cells
Source: EJNMMI Res. 2023 Dec 15;13:107. doi: 10.1186/s13550-023-01057-3 (PMC10724103; doi:10.1186/s13550-023-01057-3)
Supplement: Supplementary file 1 — Additional file 1. Supplementary methods: Materials and methods used for plasma meabolites analysis, Bio-Layer Interferometry and quantification for frozen section autoradiography. [file 13550_2023_1057_MOESM1_ESM.docx]

**Supplementary methods**

**Plasma metabolites analysis**

The in vitro stability test was performed with 40 MBq of [^18^F]Z_DGCR2:AM106_ diluted with PBS to reach a total volume of 100 µL prior to the incubation with 1 mL of human plasma at 37°C (anonymized blood samples provided by Uppsala University Hospital Blodcentralen). A sample of 300 µL was taken after 90 min for protein precipitation using acetonitrile at a 1:1 ratio. The resulting solution was then centrifuged at 16000 × g for 1 min at 4°C prior to filtering the supernatant (Acrodisc 0.2 µm Supor membrane low protein binding filter). The resulting filtrate was then analyzed using HPLC (Hitachi Chromaster HPLC system, VWR) combined with a FC-3300PMT radiodetector, a 5420 UV-VIS detector and a 5110 pump with a RP-column Vydac 214MS C5 (50 x 4.6mm). The mobile phase consisted of 10% acetonitrile in 0.1% TFA (MilliQ water) and flowed at a rate of 1 mL/min with an increasing acetonitrile gradient starting from 10% and up to 100% over 10 min. The *in vitro* plasma metabolite analysis of [^18^F]Z_DGCR2:AM106_ showed >90% intact tracer after 90 min incubation.

**Bio-Layer Interferometry (BLI)**

Ligand capture phase was performed by dipping HIS1K anti-penta-His tips (Sartorius) into PBS containing 50 µg/ml of purified recombinant human DGCR2 His-tag protein (Bio-techne; #10161-DG) for 400 s. A baseline acquisition was first performed by moving and dipping the tips into PBS for 180 s. Then the association step was evaluated by dipping the ligand coated tips into a PBS solution containing 100 nM of TCO-Z_DGCR2:AM106_ for 200 s and finally the dissociation step was in PBS studied for 400 s. All steps used 100 rpm mixing and all bio-layer interferometry experiments were performed on an OctetRED 96 (Forté BIO).

**Frozen section autoradiography quantification**

The resulting digital image readout of the frozen section pellets of Hek293 cells, endocrine pancreatic islet fractions and SC-islets, in addition to the droplet of reference radioactive solution were first delineated via manual segmentation using ImageJ. The Raw integrated density (sum of pixel values) was obtained using the “Analyze” function of the software for the delineated digital image of each sample and subtracted for background. The RawIntDen of the reference radioactive solution was equated to the cross-measured activity value from the NaI well counter. The activity of the frozen section pellets of Hek293 cells, endocrine pancreatic islet fractions and SC-islets could therefore be determined via the reference standard.

Lastly, the bound signal (BS) expressed in Bq/mm^3^ could be obtained with the following equation: BS = A/V

Where A = activity and V = volume of the section (= surface x thickness)
