# Supplementary figures and images for: Preclinical evaluation of Affibody molecule for PET imaging of human pancreatic islets derived from stem cells
Source: EJNMMI Res. 2023 Dec 15;13:107. doi: 10.1186/s13550-023-01057-3 (PMC10724103; doi:10.1186/s13550-023-01057-3)

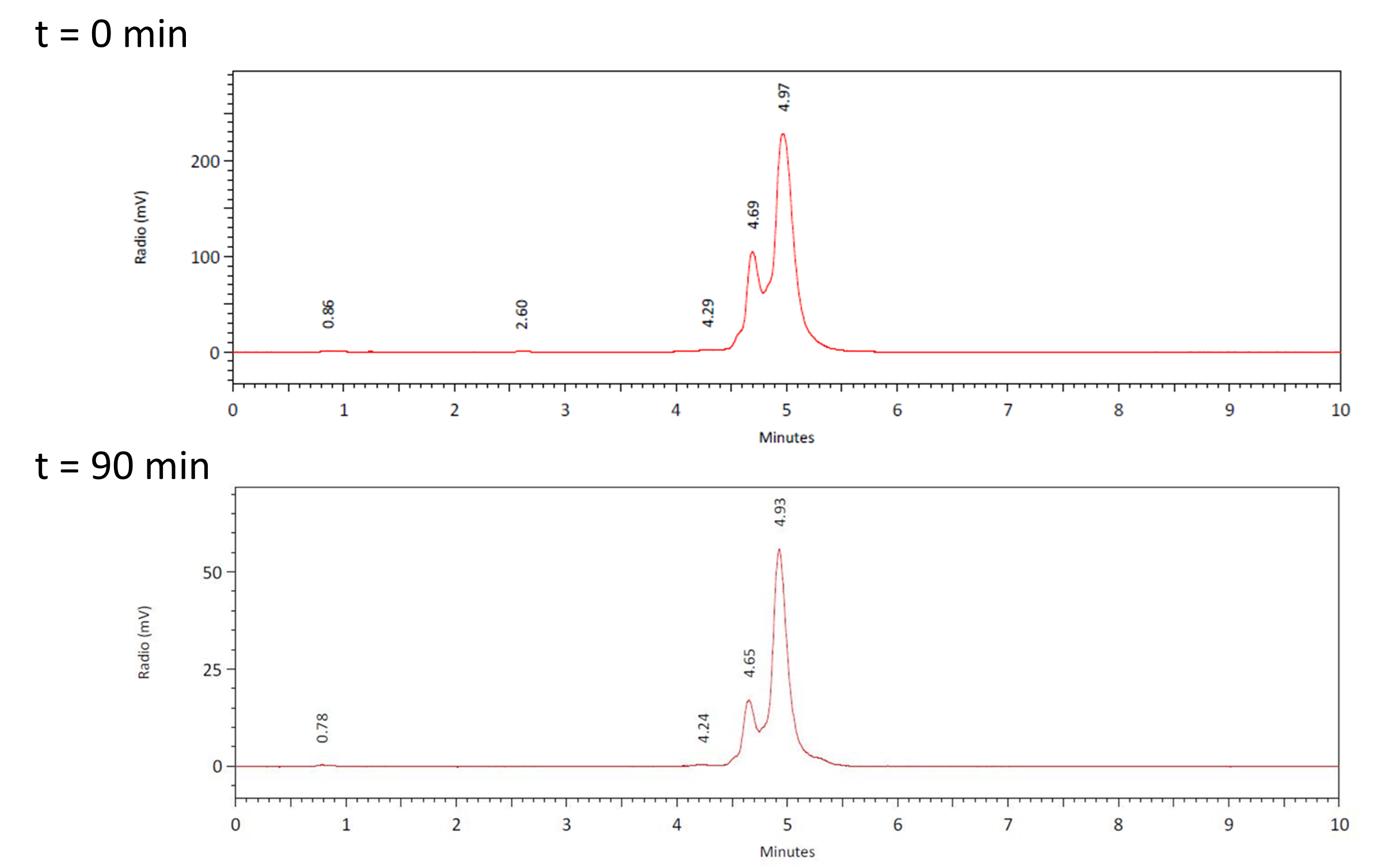

Supplement: Supplementary file 2 — Additional file 2. Fig S1: In vitro stability of [18F]ZDGCR2:AM106 in human plasma at t = 0 min and t = 90 min. [file 13550_2023_1057_MOESM2_ESM.tif]

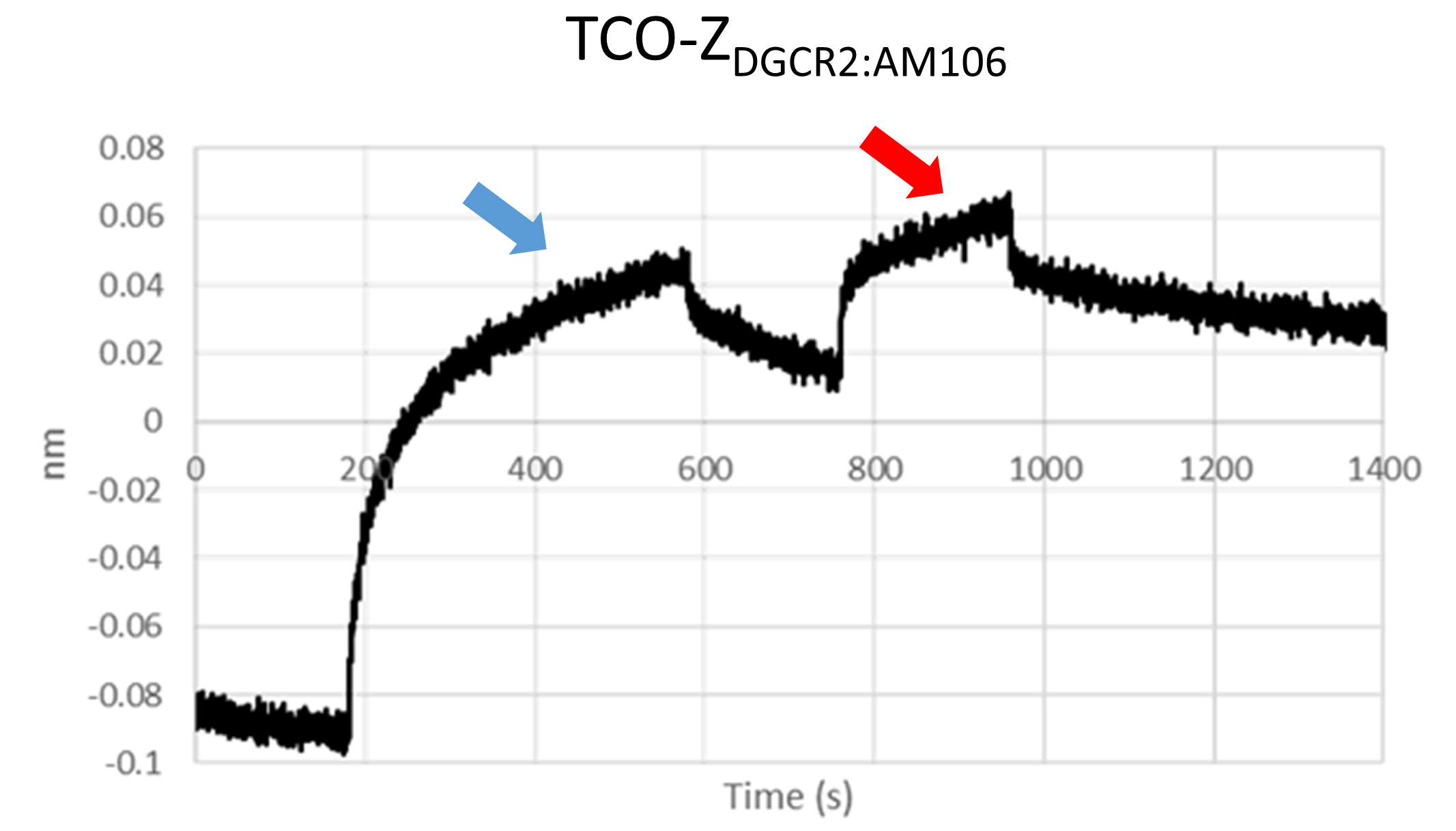

Supplement: Supplementary file 3 — Additional file 3. Fig S2: Bio-Layer interferometry result of TCO-ZDGCR2:AM106 at 100 nM. Blue arrow indicates binding of DGCR2 to the interferometer, red arrow indicates binding of TCO-ZDGCR2:AM106 interferometer. [file 13550_2023_1057_MOESM3_ESM.tif]
